# Establishment of the Complete Closed Mesh Model of Rail-Surface Scratch Data for Online Repair

**DOI:** 10.3390/s20174736

**Published:** 2020-08-21

**Authors:** Yanbin Guo, Lulu Huang, Yingbin Liu, Jun Liu, Guoping Wang

**Affiliations:** 1 Hubei Bioinformatics & Molecular Imaging Key Laboratory, Department of Biomedical Engineering, College of Life Science and Technology, Huazhong University of Science and Technology, Wuhan 430074, China; guoyanbin@hust.edu.cn (Y.G.); yipiaojun@foxmail.com (L.H.); liuyingbin@hust.edu.cn (Y.L.); 2 School of Power and Mechanical Engineering, Wuhan University, Wuhan 430072, China; liu_jun@whu.edu.cn

**Keywords:** automatic rail-surface-scratch recognition and computation, triangulation algorithm, complete closed mesh model, online rail-repair

## Abstract

Rail surface scratching occurs with increasing frequency, seriously threatening the safety of vehicles and humans. Online repair of rail-surface scratches on damaged rails with scratch depths >1 mm is of increased importance, because direct rail-replacement has the disadvantages of long operation time, high manpower and high material costs. Advanced online repair of rail-surface scratch using three-dimensional (3D) metal printing technology such as laser cladding has become an increasing trend, desperately demanding a solution for the fast and precise establishment of a complete closed mesh model of rail-surface scratch data. However, there have only been limited studies on the topic so far. In this paper, the complete closed mesh model is well established based on a novel triangulation algorithm relying on the topological features of the point-cloud model (PCM) of scratch-data, which is obtained by implementing a scratch-data-computation process following a rail-geometric-feature-fused algorithm of random sample consensus (RANSAC) performed on the full rail-surface PCM constructed by 3D laser vision. The proposed method is universal for all types of normal-speed rails in China. Experimental results show that the proposed method can accurately acquire the complete closed mesh models of scratch data of one meter of 50 Kg/m-rails within 1 min.

## 1. Introduction

In recent years, the railway industry has developed rapidly in China. The frequency and degree of rail rolling have increased sharply, and rail-surface scratches have become more and more aggravated [1,2]. A rail-surface scratch is a common rail defect caused by the metal plastic deformation due to the friction between wheels and rails and could lead to other surface defects such as peeling and fracture, which may seriously affect the safety of the train [3,4]. Rapid recognition and online-repair of rail-surface scratches is key to ensuring rails remain in good working condition, which is of great importance to safety maintenance in the railway industry [5,6,7]. 

According to the provisions of the TG/GW102-2019 in China [8], the existing ways to deal with damaged rails are presented below. Grinding [9,10] and welding [11,12] are the regular maintenance methods for rails with surface scratch depths of less than 1 mm. However, the direct replacement of the damaged rail with a brand-new one is mandatory according to the provisions mentioned above when the surface scratch depth is larger than 1 mm, which is a complicated engineering undertaking with the disadvantages of long operation time, high manpower and material costs, seriously restricting the rapid progress of the railway industry.

Recently, laser cladding, as a novel metal additive manufacturing technology, has achieved marked progress [13,14], showing the capability of repairing damaged rails online [15,16,17,18,19,20] with the advantages of convenience, high quality of the repair layer and low cost compared with the traditional replacement method. However, the precondition for online rail-repair using the laser cladding technology is to acquire the complete closed mesh model of the scratch data. The existing related works focus mostly on the detection of the damaged rail surface. Min et al. [21] and Wei et al. [22] proposed a rail surface detection system based on machine vision. Jang et al. [23] Shang et al. [24] Faghih-Roohi et al. [25] and Song et al. [26] detected the rail surface damage by using the deep learning methods. In recent years, measurement methods based on 3D laser vison have become widely used for rail surface detection. Zhimin et al. [27] proposed a 3D laser profiling system for detecting the defects on rail surface which integrated a laser scanner, odometer, inertial measurement unit (IMU) and global position system (GPS). Wang et al. [28] presented a novel method based on the local affine invariant feature descriptor for the calibration of distorted profiles obtained by traditional rail measurement system. Cao et al. [29] proposed a defect inspection method of rail surface based on the line-structured light. Santur et al. [30] described a computer-vision based approach allowing for a fast-contactless detection of the rail surface and lateral defects such as fracture, scouring and wear with high accuracy. However, so far very few studies have reported methods for establishing a complete closed mesh model of scratch data which is the precondition of online rail-repair as mentioned above.

**Figure 1:**
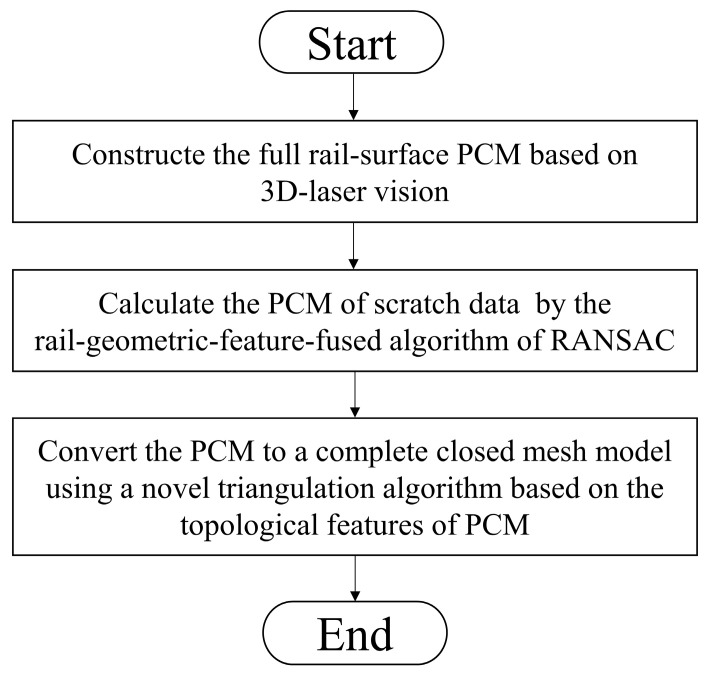
Flowchart of the proposed method.

In this paper, a method for establishing a complete closed mesh model of the rail-surface scratch data is presented. The process of the method is shown in Figure 1.

The main contributions of this paper are presented below:

(1) A systematic procedure based on a homemade 3D-laser vision system for constructing the PCM is developed.
(2) An algorithm for calculating the scratch-data PCM is presented. The algorithm is based on the RANSAC [31] fused with rail-geometric-features.
(3) A novel triangulation algorithm based on the topological features of the PCM is described. The triangulation algorithm can convert the scratch-data PCM to the complete closed mesh model required for the online rail-repair by the laser cladding technology.
(4) Experiments for verifying the proposed method are carried out. Experimental results show that our method performs well for the acquisition of a complete closed mesh model of the rail-surface scratch data.

The rest of this paper is organized as follows: Section 2 introduces the 3D-laser vision-based procedure for constructing the PCM. Section 3 describes the algorithm for calculating the scratch-data PCM. Section 4 presents the triangulation algorithm based on the point-cloud topology. Section 5 explains the experiments for verifying the proposed method. Section 6 discusses the experimental results. Finally, Section 7 draws the conclusions of the study.

## 2. 3D Modeling of Rail Surface

**Figure 2:**
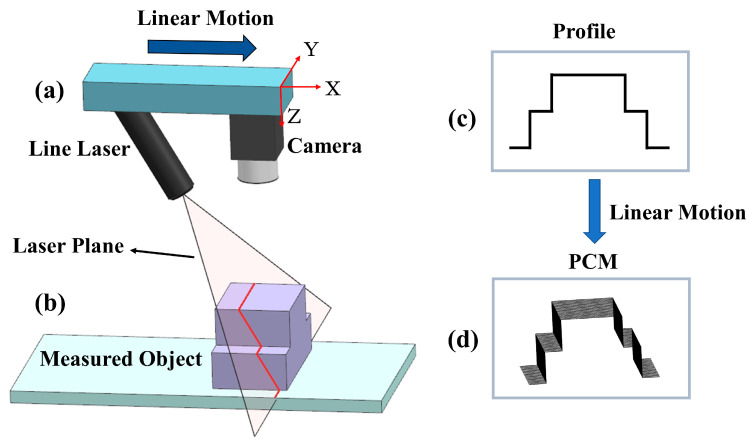
The principle of 3D laser vision. (**a**) The measurement module comprising the camera and the laser; (**b**) The laser line on the measured object located on the laser plane; (**c**) The single point-cloud profile obtained by the calculation using Equations (1) and (2); (**d**) The PCM formed with a series of point-cloud profiles schemed as in (**c**).

The principle of the 3D laser vision adopted in our paper is shown in Figure 2. The laser line moves linearly along the measured object and scans its surface, simultaneously acquiring the point cloud. The details of the procedure are described below.

In order to acquire the single point-cloud profile of the measured object, the calibration is first performed on the laser vision system. The camera model is given by the following equation:

(1) $$Z_{C}\left[\begin{array}{c} u\\ v\\ 1 \end{array}\right]=K\left[\begin{array}{c} X_{C}\\ Y_{C}\\ Z_{C} \end{array}\right]\;\mathrm{with}\;K=\left[\begin{array}{ccc} f_{x} & 0 & c_{x}\\ 0 & f_{y} & c_{y}\\ 0 & 0 & 1 \end{array}\right]$$

where (u,v) is the pixel coordinates in the pixel coordinate system, and $\left(X_{C},Y_{C},Z_{C}\right)$ is the 3D coordinates in the camera coordinate system (C-CS). The parameters in matrix K are obtained by the classical calibration method described in [32]. Distortion parameters are ignored since a distortionless camera [33] is used in this research.

Additional constraints imposed on the laser line must be considered, since Equation (1) is not sufficient for calculating the 3D coordinates. All points on the laser line are located on the laser plane as shown in Figure 2b, thus the points on the laser line in the C-CS satisfy:

(2) $$aX_{C}+bY_{C}+cZ_{C}+d=0$$

where a, b, c and d are the parameters of the laser plane equation in the C-CS, which can be obtained by the planar target calibration method [34].

After the above calibration of the laser vision system, the 3D coordinates in the C-CS of a single point-cloud profile of the measured object (Figure 2c) can be calculated using Equations (1) and (2). A series of point-cloud profiles corresponding to camera frames can also be acquired when the measurement module comprising the camera and the laser (Figure 2a) moves from the origin of the world coordinate system at a constant speed, v, and samples 1 frame every t seconds along the X-axis of the C-CS. The 3D coordinates $\left(X_{w},Y_{w},Z_{w}\right)$ in the world coordinate system can be converted from the ones in the C-CS as follows:

(3) $$\left[\begin{array}{c} X_{w}\\ Y_{w}\\ Z_{w} \end{array}\right]=\left[\begin{array}{c} X_{ci}\\ Y_{ci}\\ Z_{ci} \end{array}\right]+\left[\begin{array}{c} vti\\ 0\\ 0 \end{array}\right]$$

where $\left(X_{ci},Y_{ci},Z_{ci}\right)$ is the 3D coordinates of frame i in the C-CS. The 3D PCM of the entire measured object is acquired after the above operations (Figure 2d).

## 3. Calculation of the Scratch Data PCM

The first step of acquiring the scratch-data PCM is to accurately recognize the scratch-area of the rail surface PCM, which can differentiate between the damaged and undamaged areas. In this paper, a novel algorithm for the scratch-recognition on rail surface is proposed by combining RANSAC with the geometric features of the rail profile. 

**Figure 3:**
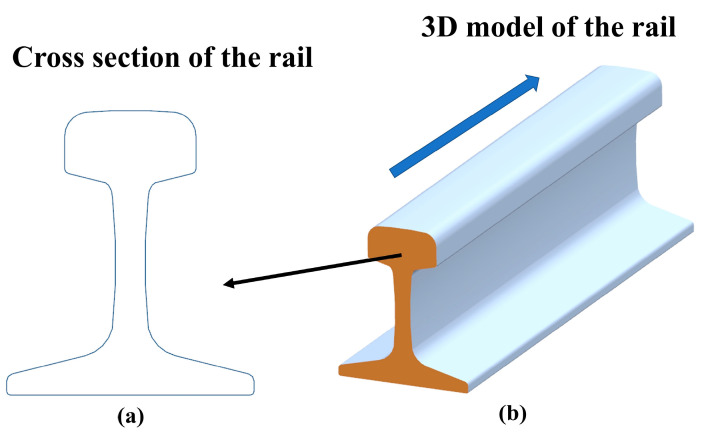
The geometry of the undamaged rail. (**a**) The cross section of the rail; (**b**) 3D model of the rail.

According to the hot-rolled rails for railway technology standard GB2585-2007 [35], the geometry of all types of the original undamaged rail can be illustrated as Figure 3. The fixed geometry of the cross section (Figure 3a) can be extended in the direction perpendicular to itself to form the entire rail (Figure 3b). 

**Figure 4:**
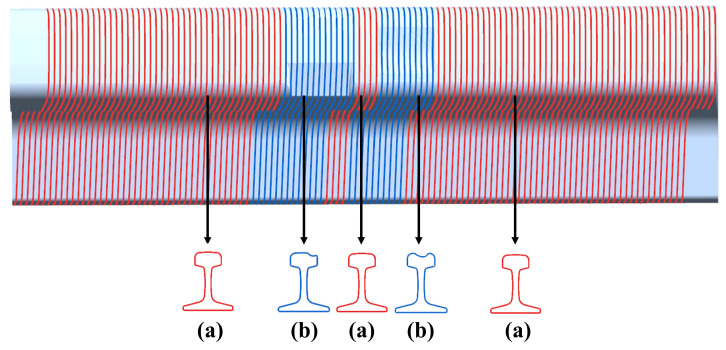
Geometric features of the rail profiles. (**a**) The point-cloud profiles of the undamaged areas with the same geometric features; (**b**) The point-cloud profiles of the damaged areas with a variety of shapes and no uniform geometric features.

As represented in Figure 4, the PCM obtained by 3D scanning of the physical rail using the procedure described in Section 2 comprises a series of equidistant point-cloud profiles. An analysis reveals that all the point-cloud profiles corresponding to the undamaged portions of the rail-surface have the same geometric features as illustrated in Figure 4a, but the ones corresponding to the damaged areas have varying shapes and no uniform geometric features as indicated in Figure 4b. Thus, the point-cloud profiles corresponding to the undamaged areas can be easily and accurately filtered out by using the above observation, resulting in the recognition of scratch-areas.

**Figure 5:**
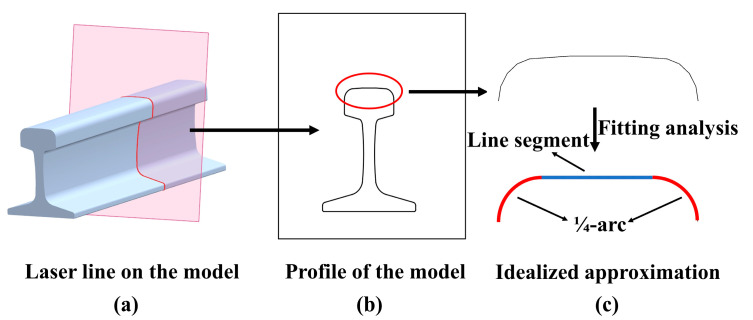
The uniform geometry of the laser profile of the undamaged rail. (**a**) The laser line on the model; (**b**) The single profile acquired from (**a**); (**c**) The idealized approximation of the profile indicated with the red circle in (**b**).

The mathematical method based on the RANSAC fused with the geometric features of the rail for filtering out the point-cloud profiles on undamaged areas is described as follows. Figure 5 presents an idealized approximation of the geometric features (Figure 5a, Table S1b) by fitting a line segment and two ¼-arcs of the same radii (Figure 5c) using the RANSAC algorithm. A subset of points from the entire point-cloud profile of the rail are randomly selected as the start of RANSAC flow. The selected point-subset is fitted to mathematical models using a least square’s procedure to obtain preliminary model parameters, which are subsequently used to calculate the deviation of all 3D points in the point-cloud profile. If the deviation is less than a predetermined threshold, the 3D point is classified to an inlier, otherwise, an outlier. After performing a finite number of iterations of the aforementioned process, the model parameters corresponding to the largest number of inliers can be selected as the best model parameter estimates [36].

**Figure 6:**
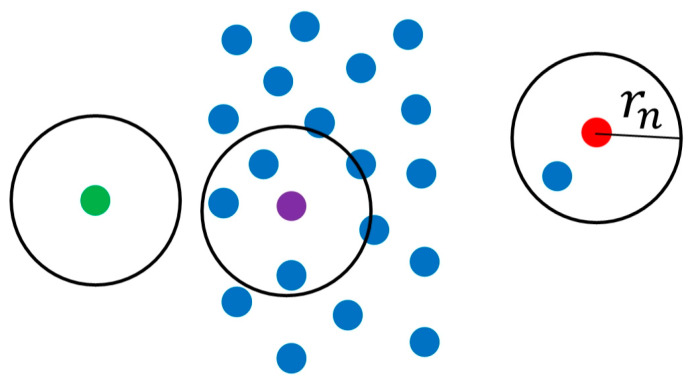
The point cloud filtering method based on the neighborhood radius.

**Figure 7:**
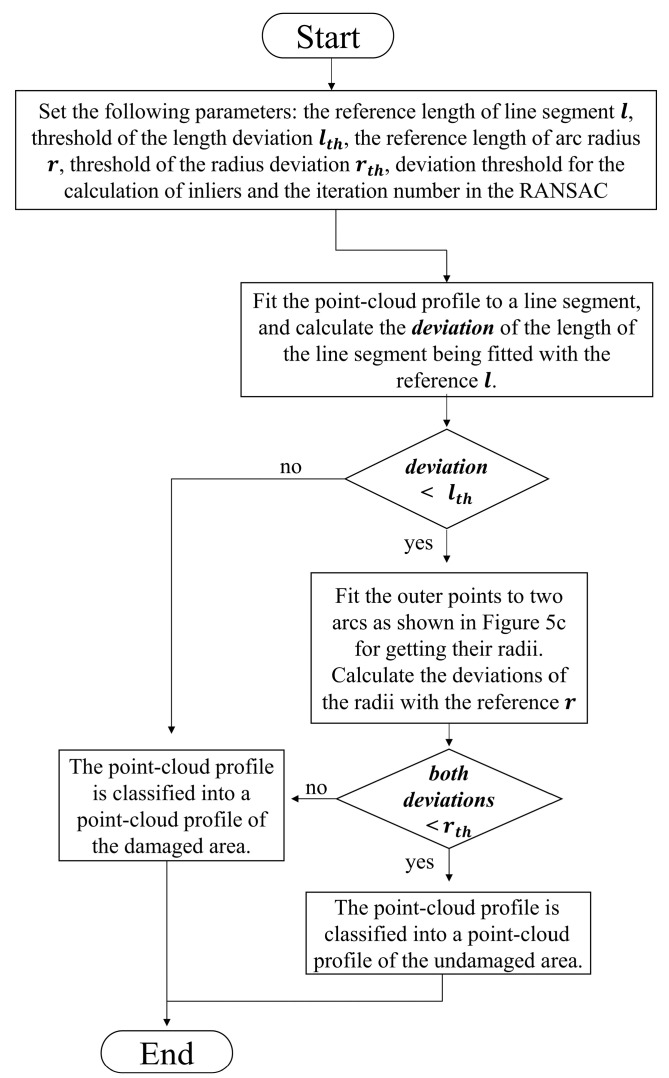
Flowchart of the method for classifying the point-cloud profiles.

**Figure 8:**
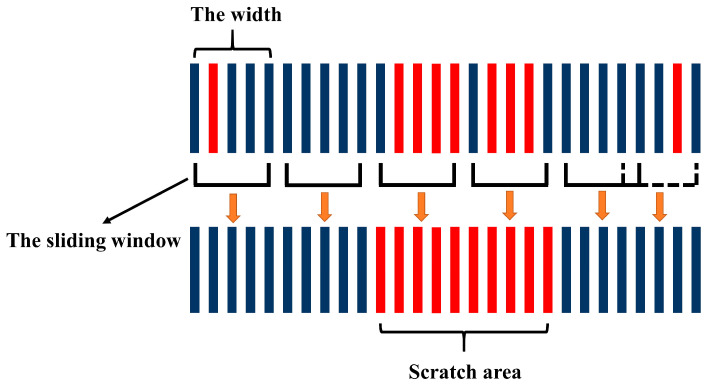
The method for re-classifying profiles by the sliding window with a certain threshold width. The red lines represent the profiles in scratched areas and the blue lines represent the profiles in undamaged areas.

In order to recognize the scratch-area of the rail surface PCM, the algorithm is developed based on the above mathematical method as follows:

(1) Filter the rail surface PCM to remove the noise points and outliers for improving its quality [37] by the method based on the neighborhood radius as shown in Figure 6. Set the radius of the neighborhood as $r_{n}$ and the minimum number of points in a neighborhood as n. If the minimum number of points in a neighborhood with the radius of $r_{n}$ is less than n, the point will be filtered out. As shown in Figure 6, the red point and the green point will be filtered out when n=2.
(2) Split the rail surface PCM into a series of point-cloud profiles and process them separately by the method presented in Figure 7, classifying them into the damaged area or undamaged area, respectively.
(3) Recognize the scratch-area of the PCM based on the above classification result. A few point-cloud profiles may be not classified correctly, because of errors caused by various reasons, and should be restored as follows. The method shown in Figure 8 is proposed based on the knowledge that the damaged area and the undamaged area on the rail-surface PCM are consecutive within a certain width range. When the sliding window with a certain width scans the profiles of different classified areas, if the majority of profiles belong to the damaged area, then the minority will be re-classified into the damaged area and vice versa as displayed in Figure 8.

The scratch-surface PCM can be accurately acquired by recognizing the scratch area of the rail-surface PCM with the above-mentioned algorithm, which is subsequently used to calculate the difference with the reference PCM constructed by the method described below.

According to the geometry of the rail presented in Figure 3, the reference PCM can be constructed by extending the undamaged profile in the certain direction. The mathematical formulation is as follows:

(4) $$\left[\begin{array}{c} X_{SS}\\ Y_{SS}\\ Z_{SS} \end{array}\right]=\left[\begin{array}{c} X_{S}\\ Y_{S}\\ Z_{S} \end{array}\right]+\left[\begin{array}{c} x_{\Delta }\\ x_{\Delta }\frac{n_{y}}{n_{x}}\\ x_{\Delta }\frac{n_{z}}{n_{x}} \end{array}\right]$$

where $x_{\Delta }$ is the extended step length, $\vec{v}=(n_{x},n_{y},n_{z})$ is the extension vector, $\left(X_{S},Y_{S},Z_{S}\right)$ is the point of the undamaged profile, and $\left(X_{SS},Y_{SS},Z_{SS}\right)$ is the constructed point in the reference PCM.

**Figure 9:**
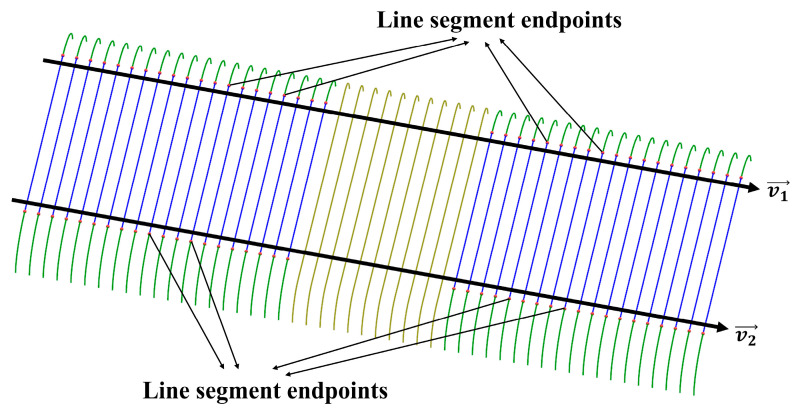
Fitting the endpoints of line segments with a model of line to acquire the preliminary extension vectors $\vec{v_{1}}$ and $\vec{v_{2}}$.

The most critical step for constructing the reference PCM is to accurately calculate the extension vector: $\vec{v}=(n_{x},n_{y},n_{z})$. In Figure 9, the point-cloud profiles on unscratched region are approximated with a line segment of equal length and two 1/4 arcs of equal radii as described previously. The two sets of corresponding endpoints of line segments are fitted respectively with a model of line whose direction is defined as the preliminary extension vector. Thus, two preliminary vectors, $\vec{v_{1}}=(n_{x1},n_{y1},n_{z1})$ and $\vec{v_{2}}=(n_{x2},n_{y2},n_{z2})$ are obtained for the calculation of final extension vector, $\vec{v}$, by using the following equation:

(5) $$\vec{v}=\left[\begin{array}{c} n_{x}\\ n_{y}\\ n_{z} \end{array}\right]=\theta_{1}\left[\begin{array}{c} n_{x1}\\ n_{y1}\\ n_{z1} \end{array}\right]+\theta_{2}\left[\begin{array}{c} n_{x2}\\ n_{y2}\\ n_{z2} \end{array}\right]\;with\;\theta_{1}+\theta_{2}=1$$

where $\theta_{1}$ and $\theta_{2}$ are the weighting parameters.

After the final extension vector $\vec{v}=(n_{x},n_{y},n_{z})$ is acquired, the reference PCM can be constructed. Finally, the differences between the reference PCM and the scratch-surface PCM can be calculated and the PCM pair with a difference larger than a certain threshold will be selected to form the PCM of scratch-data.

## 4. 3D Point-Cloud Triangulation

The PCM of scratch data obtained in Section 3 cannot be used directly for the online rail-repair relying on the laser cladding technology since it is not a complete closed mesh model and does not meet the requirements for layering of 3D printing [38]. In order to quickly and efficiently achieve a complete closed mesh model, a novel triangulation-algorithm [39] based on the topological features of the PCM is proposed in this section. 

**Figure 10:**
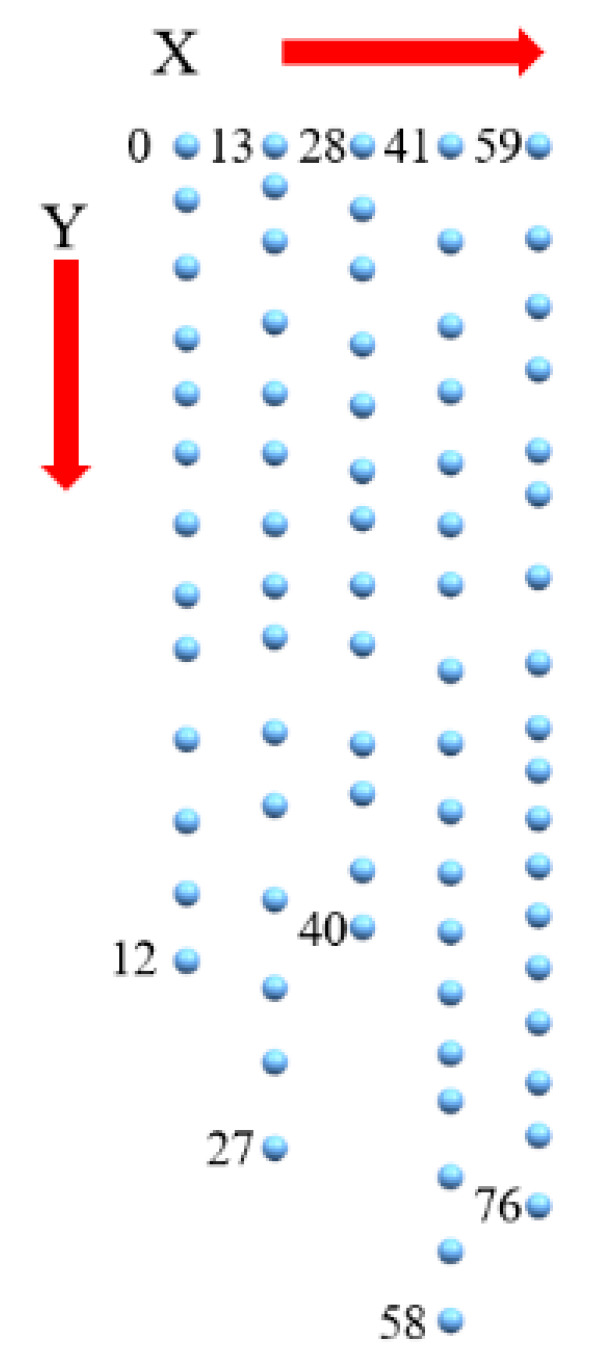
The topological features of the 3D surface PCM labelled with the index numbers.

**Figure 11:**
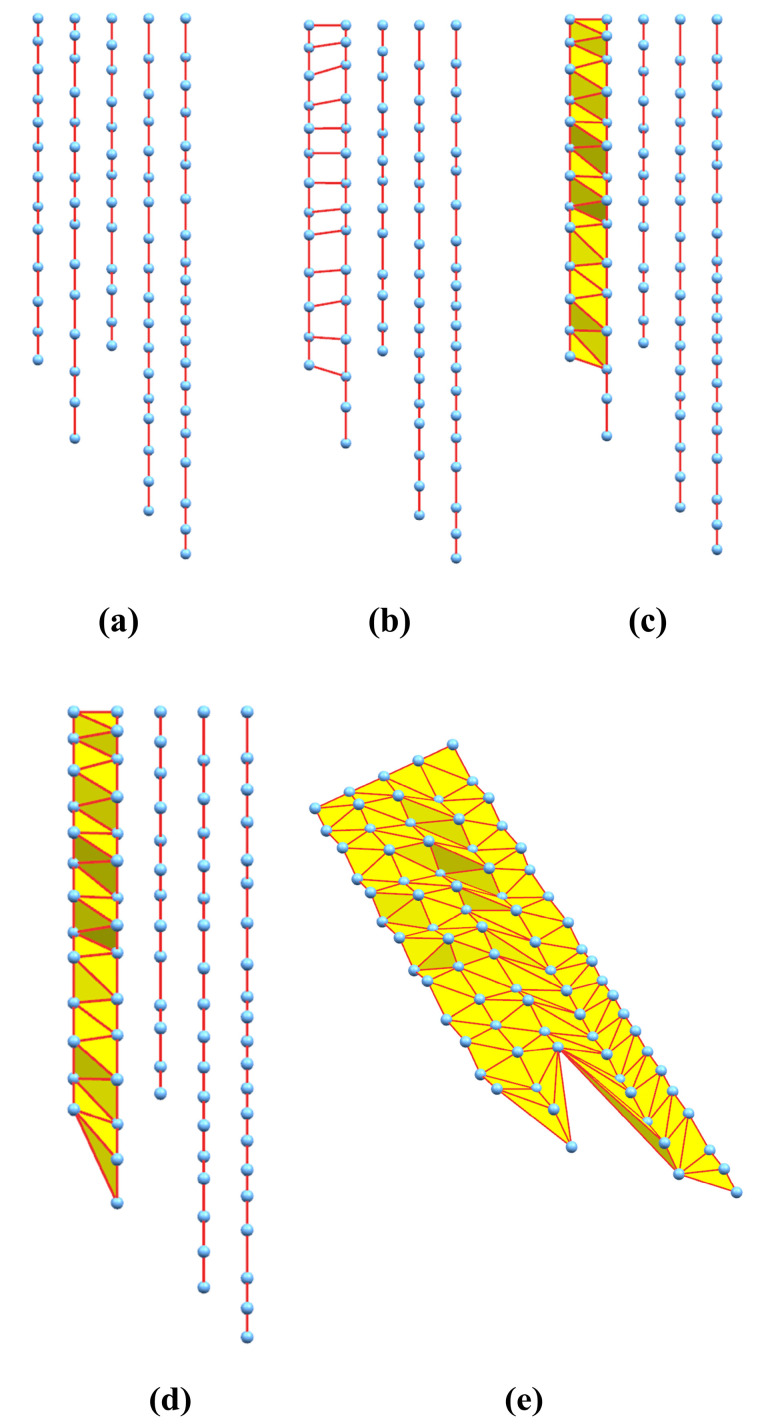
The schematized flow of the triangulation algorithm of 3D point-cloud. (**a**) Concatenate each point-cloud profile with lines; (**b**) Constructe the quadrilaterals; (**c**) Form the triangle-meshes; (**d**) Finish the triangulation of the adjacent point-cloud profiles; (**e**) Complete the triangulation algorithm for the PCM.

Firstly, filtering of the PCM is performed to remove the noise points and outliers caused by the calculation errors using the method presented in Figure 6. Secondly, the filtered-PCM is decomposed into a series of line-profiles equidistant in the X-axis direction since the obtained PCM has an ordered structure (Figure 10) according to the 3D modeling procedure proposed in Section 2. Finally, the triangulation algorithm is implemented as illustrated in Figure 11.

Each point-cloud profile is concatenated with lines as shown in Figure 11a. Then the triangle-meshes between the adjacent point-cloud profiles are sequentially constructed following Figure 11b–d. In Figure 11b, the quadrilaterals are constructed first, then in Figure 11c, triangle-meshes are formed within the above quadrilaterals. In Figure 11d, the remaining points in one of the point-cloud profiles are connected with the end point of the counterpart, finishing the triangulation of the adjacent point-cloud profiles. By carrying out the similar procedure described above between all adjacent point-cloud profiles, the triangulation algorithm for the PCM is completed with a result of Figure 11e.

**Figure 12:**
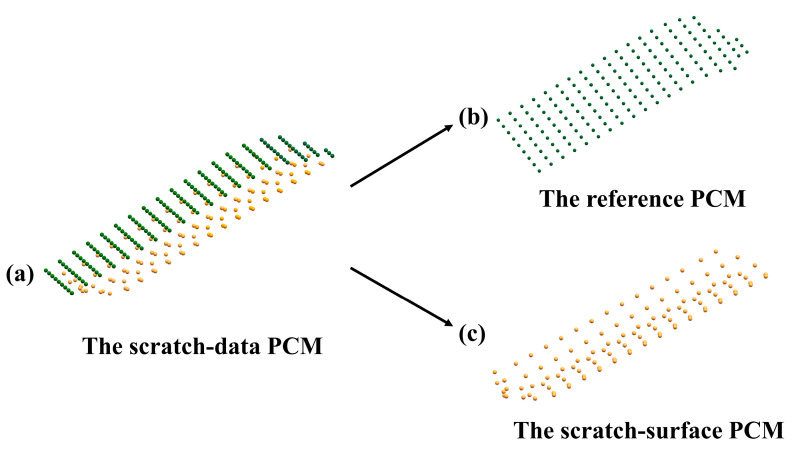
The composition of the scratch-data PCM. (**a**) The scratch-data PCM; (**b**) The reference PCM; (**c**) The scratch-surface PCM.

**Figure 13:**
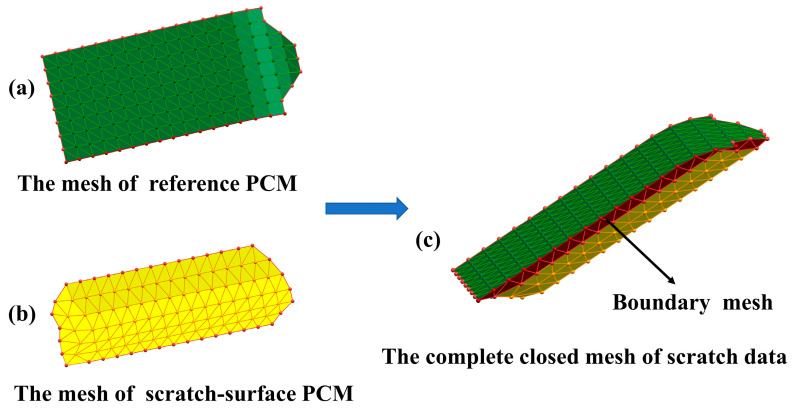
The process of constructing the complete closed mesh model of scratch data. (**a**) The mesh of reference PCM; (**b**) The mesh of scratch-surface PCM; (**c**) The complete closed mesh model of scratch data stitched by (**a**,**b**) through the boundary mesh.

Thus, the triangulation of scratch-data PCM can be done by separately triangulating the reference PCM (Figure 12b) and the scratch-surface PCM (Figure 12c) using the algorithm described above since they compose scratch-data PCM as illustrated in Figure 12a, which also can be found in Section 3. Subsequently, the final complete closed mesh model of the scratch-data (Figure 13c) can be successfully achieved by stitching the triangle-meshes of the reference PCM (Figure 13a) and the scratch-surface PCM (Figure 13b) through the boundary mesh as schemed in Figure 13.

## 5. Experiment

Experiments are carried out on both the artificial rail and a practical rail to verify the proposed method. All algorithms are run on a computer whose specifications are listed in Table 1. 

The homemade 3D laser vision system proposed in Section 2 is used for constructing the PCM of the rail-surface. The main specifications of the line laser and the camera used in the system are listed in Table 2 and Table 3 respectively. The parameters in the system mentioned in Section 2 are listed in Table 4.

**Figure 14:**
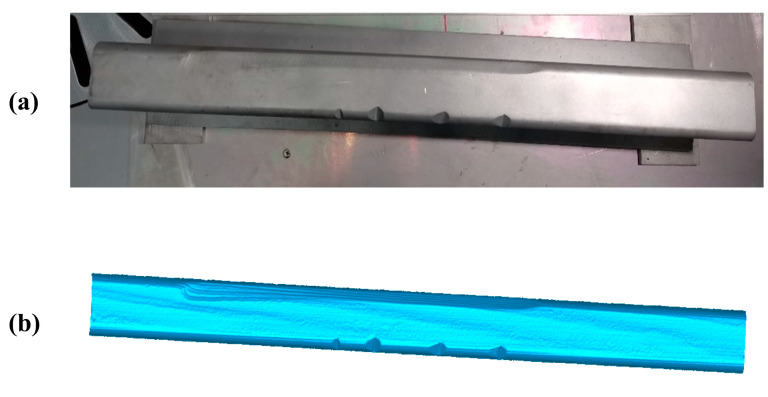
The artificial rail of 50 Kg/m and its surface PCM. (**a**) The artificial damaged-rail; (**b**) The surface PCM of the artificial damaged-rail.

The artificial 50 Kg/m-rail presented in Figure 14a is used in the first experiment. The length of the rail is 1 m, the scanning time for obtaining the data is 1/0.04 = 25 s and the time of data processing to form the 3D PCM of the rail-surface (Figure 14b) is 10.63 s.

**Figure 15:**
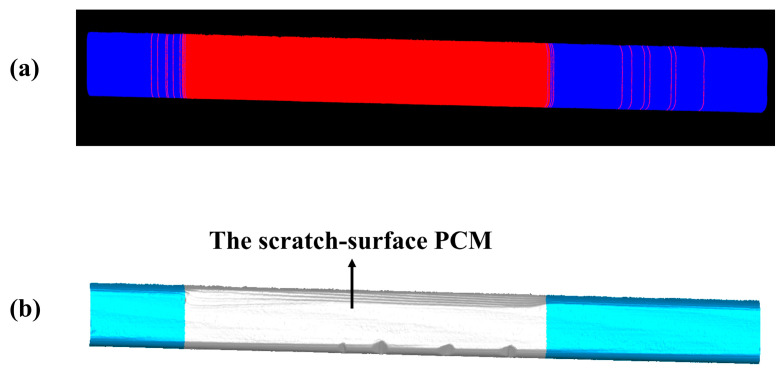
The result of the scratch-recognition algorithm performed on the rail-surface PCM. (**a**) The classification result of the point-cloud profiles displaying the damaged area (red) and the undamaged area (blue); (**b**) The scratch-surface PCM identified by the algorithm as indicated in the gray-white area.

The scratch-recognition algorithm presented in Section 3 is performed on the PCM of the rail-surface. There is no need to carry out the filtering algorithm because of the fine quality of the PCM as shown in Figure 14b. A series of point-cloud profiles are generated by splitting the rail-surface PCM and subsequently processed separately following the method described in Figure 7 with the values of the parameters listed in Table 5. The classification result of the point-cloud profiles is reflected in Figure 15a, where the red profiles and the blue profiles are classified into the damaged area and the undamaged area, respectively. A few point-cloud profiles incorrectly classified are restored with the method expressed in Figure 8 in which the width of the sliding window is set as 5 profiles. Figure 15b indicates the scratch-surface PCM which is the final result of the scratch-recognition algorithm with the total running time of 1.27 s.

The reference PCM can easily be acquired by extending an undamaged point-cloud profile selected from the rail-surface PCM using the Equation (4) with the extending step length $x_{\Delta }=0.5$ mm. The extension vector in Equation (4) can be calculated from preliminary vectors (see Figure 9) using the Equation (5) with $\theta_{1}$ = 0.5, $\theta_{2}$ = 0.5. Table 6 displays the calculation results of the parameters mentioned here.

**Figure 16:**
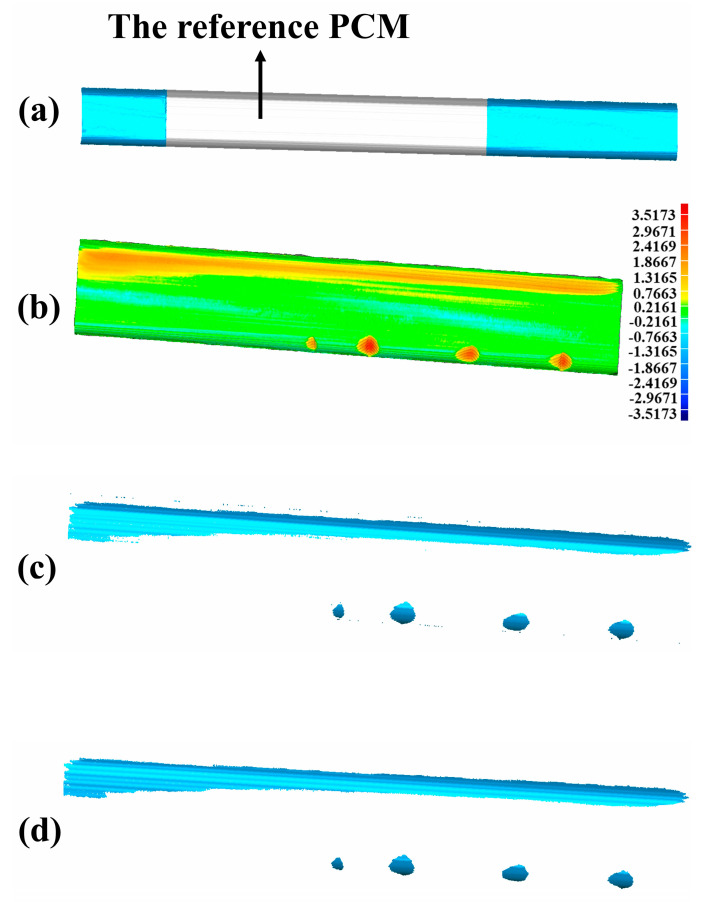
The acquisition of the scratch-data PCM. (**a**) The result of constructed reference PCM; (**b**) The depth-difference between the reference PCM and the scratch-surface PCM; (**c**) The original scratch-data PCM with noise points; (**d**) The filtered scratch-data PCM.

After the above calculations, the reference PCM is constructed as seen in Figure 16a. Then, the depth-difference between the reference PCM and the scratch-surface PCM shown in Figure 16b is calculated. If the depth-difference is larger than the set threshold of 0.2 mm, the reference PCM and the scratch-surface PCM are selected to construct the original scratch-data PCM shown in Figure 16c. The total time for acquiring the scratch-data PCM starting from the reference PCM construction is 3.23 s.

**Figure 17:**
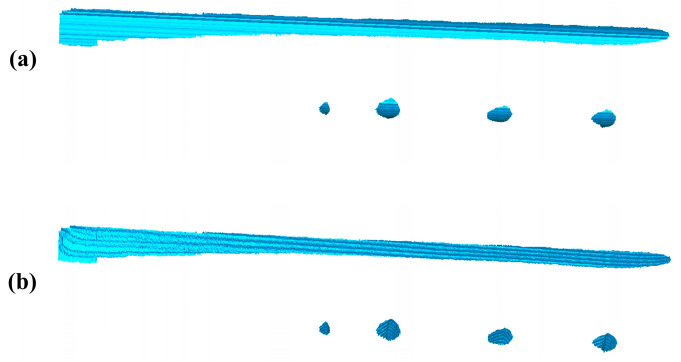
The triangulation of the PCM. (**a**) The triangle-meshes of the reference PCM; (**b**) The triangle-meshes of the scratch-surface PCM.

**Figure 18:**
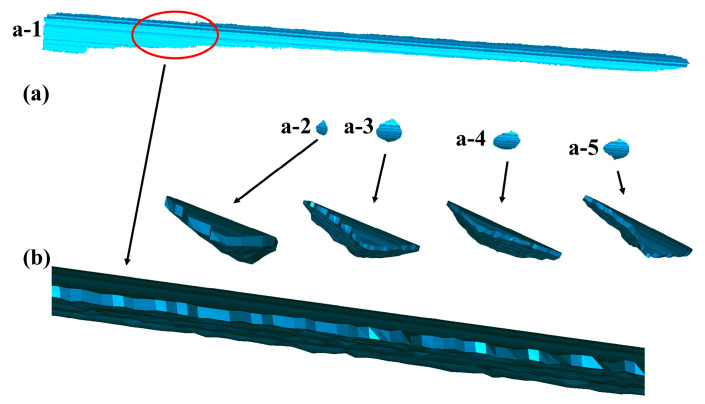
The final complete closed mesh models of the scratch-data of the artificial damaged rail. (**a**) Five complete closed mesh models corresponding to five scratch-data; (**b**) The local magnified model of a−1 and the full magnified ones of a−2, a−3, a−4, a−5, respectively.

The 3D triangulation-algorithm proposed in Section 4 is performed on the scratch-data PCM. The filtering of the PCM is firstly done before the formal triangulation based on the method of neighborhood radius presented in Figure 6 with $r_{n}$ = 4 mm and n = 50, leading to the result of Figure 16d. Then, the triangulation of the reference PCM (Figure 17a) and the scratch-surface PCM (Figure 17b) are finished by using the algorithm presented in Figure 11. Finally, a complete closed mesh model required for laser cladding technology as shown in Figure 18a (the magnified details presented in Figure 18b) is well established by stitching the triangle-meshes of the reference PCM and the scratch-surface PCM through the boundary mesh, which is the end of the whole experiment. The triangulation-algorithm costs 5.22 s in our experiment. Table 7 lists the time required for each step in the experiment and the total time is 45.35 s. 

**Figure 19:**
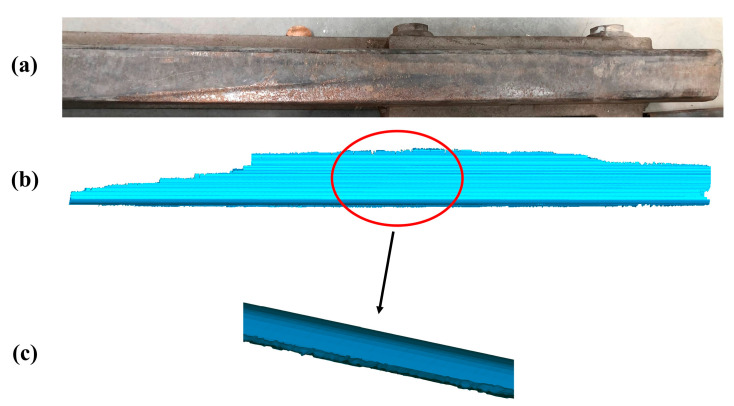
The practical damaged rail and final result in the second experiment. (**a**) The practical damaged-rail; (**b**) The final complete closed mesh model of the practical damaged rail; (**c**) The local magnified model of b.

The second experiment is carried out on the practical 50 Kg/m-rail presented in Figure 19a to further verify the reliability of the proposed method. The final complete closed mesh model of the practical damaged rail is established by the same method described in the first experiment, which is shown in Figure 19b (the magnified details presented in Figure 19c). The total time for the second experiment is 47.51 s. The detailed process of the second experiment which is similar to the one presented above for the artificial rail, is well described with Figures S1–S5 and Tables S1–S3 included in the supplementary materials.

## 6. Discussion

**Figure 20:**
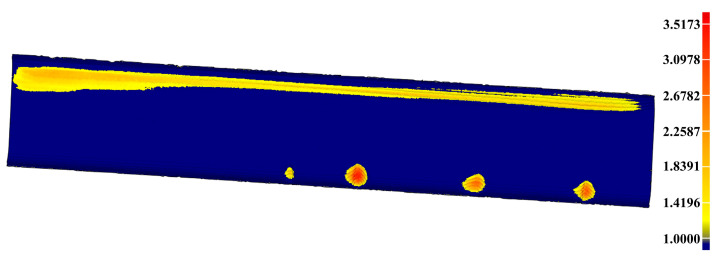
The specific places on the artificial rail surface with scratch-depth larger than 1 mm.

**Figure 21:**
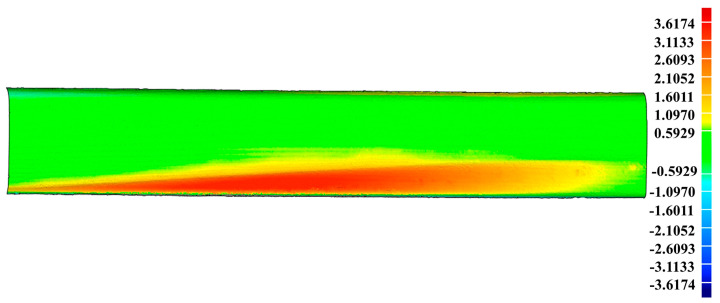
The depth-difference between the reference PCM and the scratch-surface PCM on the practical damaged rail.

**Figure 22:**
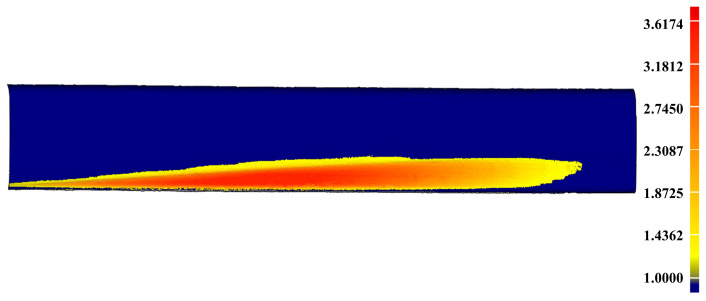
The specific places on the practical damaged rail surface with scratch-depth larger than 1 mm.

In the first experiment for the artificial damaged rail, Figure 16b obviously shows that the scratch-depth of most rail surface is larger than 1 mm and the specific places of scratch-depth > 1 mm is presented in Figure 20. Table 8 displays the further analysis result in the form of max depth, min depth and Root Mean Square (RMS) acquired by Geomagic Studio 2014 (64 bit). The counterpart results in the second experiment for the practical damaged rail are presented in Figure 21, Figure 22, and Table 9, respectively. Based on the above results, both the artificial rail and the practical rail in the experiment should be replaced with new rails, which is mandatory required by the provisions of the TG/GW102-2019 in China.

**Figure 23:**
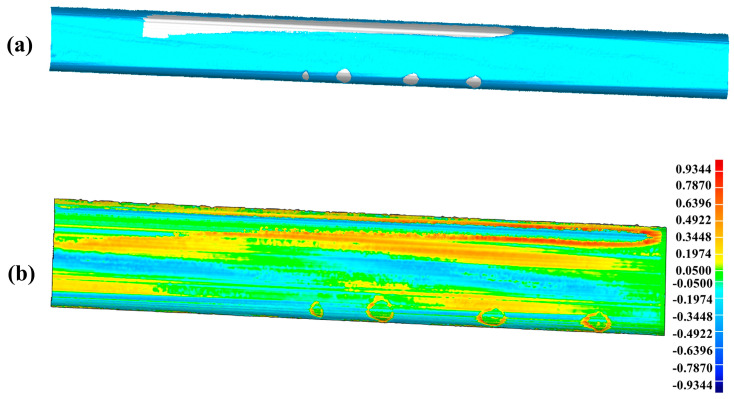
The accuracy analysis of the scratch-data acquired in the experiment for the artificial damaged rail. (**a**) The result of virtual repair of the artificial rail by using the scratch-data; (**b**) The difference between the repaired artificial rail model and the reference model indicating the scratch-depth on the repaired artificial rail is less than 1 mm.

**Figure 24:**
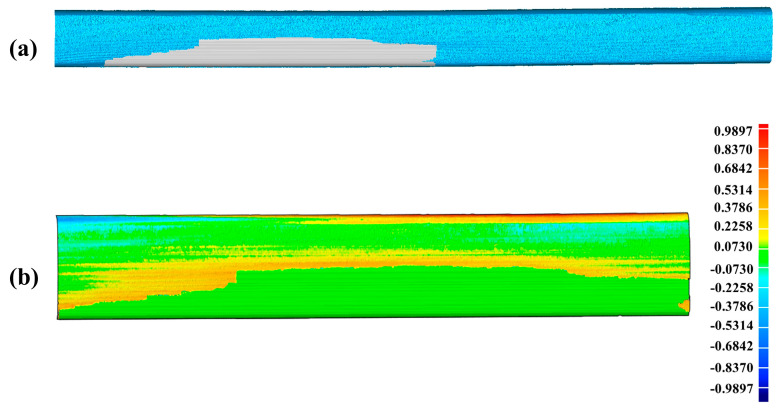
The accuracy analysis of the scratch-data acquired in the experiment for the practical damaged rail. (**a**) The result of virtual repair of the practical rail by using the scratch-data; (**b**) The difference between the repaired practical rail model and the reference model indicating the scratch-depth on the repaired practical rail is less than 1 mm.

In order to verify the accuracy of the complete closed mesh models of the scratch-data acquired in the experiment, the virtual repairs of the rails by cladding the scratch-area with the models are carried out, leading to the results of Figure 23a and Figure 24a, which are corresponding to the artificial rail and the practical rail, respectively. The difference calculated between the repaired artificial rail model and the reference model is shown in Figure 23b and further analyzed in Table 10. The similar results for the practical rail are presented in Figure 24b and Table 11. 

The above results show that the scratch-depth on the repaired rails is less than 1 mm, which well meets the requirements in the provisions of the TG/GW102-2019 in China, proving that the complete closed mesh model of the scratch-data established herein is precise enough for the use of online repair. Also, the total time required in the experiments (45.35 s for the artificial rail and 47.51 s for the practical one) is much less than 1 min, fully demonstrating the crucial capability of real-time required by online rail-repair based on the laser cladding technology, so the method proposed in our paper is practical for the online repairing of damaged rails in terms of accuracy and real-time performance.

## 7. Conclusions

In this paper, an efficient and accurate method is well proposed for the establishment of the complete closed mesh model of rail-surface scratch data, solving the precondition of online rail-repair based on the laser cladding technology. Related experiments are performed on both the artificial rail and the practical rail, and the corresponding results reveal the capability of real-time and accuracy required by the online rail-repair, which could further promote the development of the field.

## Figures and Tables

**Table 1:** The specifications of the computer used in the experiment.

Parameter	Value
Memory size	16 GB
CPU type	Intel Core i5-9400F
GPU type	NVIDIA RTX2060
Graphics memory size	6 GB

**Table 2:** The main specifications of the line laser.

Parameter	Value
Type	ZLM5AL650-16GD0.15
Overall dimension	16 mm × 16 mm × 70 mm
Power	5 mw
Wavelength	650 nm
Minimum line width	0.15 mm

**Table 3:** The main specifications of the camera.

Parameter	Value
Type	MV-GE134GC-T-CL
Overall dimension	29 mm × 29 mm × 40 mm
Pixel size	1,300,000
Resolution	1280 × 1024
Maximum frame rate	91 FPS

**Table 4:** The parameters in the 3D laser vision system.

Parameter	Value
The intrinsic matrix of the camera after calibration	$K=\left[\begin{array}{ccc} 691.69732 & 0 & 366.62759\\ 0 & 691.79845 & 241.07942\\ 0 & 0 & 1 \end{array}\right]$
The laser plane equation	$0.006X_{C}-1.05453Y_{C}-Z_{C}+478.982=0$
The speed of the measurement module	v=0.04 m/s
The sampling frequency of the camera	80 HZ
The sampling interval	t=1/80=0.0125 s

**Table 5:** The values of the parameters mentioned in Figure 7.

Parameter	Value
l	50 mm
$l_{th}$	1 mm
r	15 mm
$r_{th}$	1 mm
Deviation threshold in RANSAC	0.2 mm
Iteration number in RANSAC	1000

**Table 6:** The calculation results of the extension vector.

Vector	Value
$\vec{v_{1}}$	(0.999923, 0.011243, 0.005168)
$\vec{v_{2}}$	(0.999936, 0.010394, 0.004364)
$\vec{v}$	(0.999930, 0.010819, 0.004766)

**Table 7:** The time required in the experiment for the artificial damaged rail.

Time	Value
Scanning time	25 s
3D PCM constructing	10.63 s
Scratch-recognition	1.27 s
Scratch-data acquiring	3.23 s
3D triangulation	5.22 s
Total time	45.35 s

**Table 8:** The analysis result of the artificial damaged rail.

Max Depth	Min Depth	RMS
3.5173 mm	−0.7009 mm	0.5579 mm

**Table 9:** The analysis result of the practical damaged rail.

Max Depth	Min Depth	RMS
3.6174 mm	−0.7363 mm	1.0520 mm

**Table 10:** The analysis result of the repaired artificial rail.

Max Depth	Min Depth	RMS
0.9344 mm	−0.7009 mm	0.1928 mm

**Table 11:** The analysis result of the repaired practical rail.

Max Depth	Min Depth	RMS
0.9897 mm	−0.7363 mm	0.1824 mm

## Supplementary Materials

The following are available online at https://www.mdpi.com/1424-8220/20/17/4736/s1, Figure S1: The practical rail of 50 Kg/m and its surface PCM, Figure S2: The result of the scratch-recognition algorithm performed on the rail-surface PCM of the practical rail, Figure S3: The acquisition of the scratch-data PCM, Figure S4: The triangulation of the PCM, Figure S5: The final complete closed mesh models of the scratch-data of the practical rail, Table S1: The values of the parameters used in the scratch-recognition algorithm, Table S2: The calculation results of the extension vector, Table S3: The time required in the experiment.
